# The northernmost hyperspectral FLoX sensor dataset for monitoring of high-Arctic tundra vegetation phenology and Sun-Induced Fluorescence (SIF)

**DOI:** 10.1016/j.dib.2023.109581

**Published:** 2023-09-16

**Authors:** Hans Tømmervik, Tommaso Julitta, Lennart Nilsen, Taejin Park, Andreas Burkart, Katarzyna Ostapowicz, Stein Rune Karlsen, Frans-Jan Parmentier, Norbert Pirk, Jarle W. Bjerke

**Affiliations:** a Norwegian Institute of Nature Research (NINA), FRAM - High North Centre for Climate and the Environment, Tromsø 9296, Norway; b JB Hyperspectral Devices, Am Botanishen Garten 33, Düsseldorf 40225, Germany; c Department of Arctic and Marine Biology, UiT - The Arctic University of Norway, Tromsø 9037, Norway; d NASA Ames Research Center, Moffett Field, CA 94035, USA; e Bay Area Environmental Research Institute, Moffett Field, CA 940354, USA; f NORCE Norwegian Research Centre AS, P.O. Box 6434, 9294 Tromsø, Norway; g Department of Geosciences Center for Biogeochemistry in the Anthropocene, University of Oslo, Oslo 0315, Norway; h Department of Physical Geography and Ecosystem Science, Lund University, Lund 223 62, Sweden

**Keywords:** Chlorophyll fluorescence, Solar Induced Fluorescence (SIF), Reflectance, Photosynthetic function, MERIS terrestrial chlorophyll index (MTCI), High-Arctic tundra

## Abstract

A hyperspectral field sensor (FloX) was installed in Adventdalen (Svalbard, Norway) in 2019 as part of the Svalbard Integrated Arctic Earth Observing System (SIOS) for monitoring vegetation phenology and Sun-Induced Chlorophyll Fluorescence (SIF) of high-Arctic tundra. This northernmost hyperspectral sensor is located within the footprint of a tower for long-term eddy covariance flux measurements and is an integral part of an automatic environmental monitoring system on Svalbard (AsMovEn), which is also a part of SIOS. One of the measurements that this hyperspectral instrument can capture is SIF, which serves as a proxy of gross primary production (GPP) and carbon flux rates. This paper presents an overview of the data collection and processing, and the 4-year (2019–2021) datasets in processed format are available at: https://thredds.met.no/thredds/catalog/arcticdata/infranor/NINA-FLOX/raw/catalog.html associated with https://doi.org/10.21343/ZDM7-JD72 under a CC-BY-4.0 license. Results obtained from the first three years in operation showed interannual variation in SIF and other spectral vegetation indices including MERIS Terrestrial Chlorophyll Index (MTCI), EVI and NDVI. Synergistic uses of the measurements from this northernmost hyperspectral FLoX sensor, in conjunction with other monitoring systems, will advance our understanding of how tundra vegetation responds to changing climate and the resulting implications on carbon and energy balance.

Specifications Table

**Table:** 

Subject	Ecology

Specific subject area	Remote sensing monitoring of vegetation photosynthetic function, phenology, canopy reflectance, and fluorescence measurements.
Data format	Raw Processed
Type of data	Tables of contemporaneous, coupled time series of observations from the FLoX-spectrometer.
Data collection	FloX measurements: were collected from 2 m above the canopy, at the frequency defined by the system optimization program resulting in approximately 1000 measurements per day. Canopy upwelling and down-welling passive optical measurements were collected using a Dual Fluorescence boX (FloX, JB Hyperspectral Devices AG, Dusseldorf, Germany) [1] system. These spectral measurements were processed to provide reflectance and solar induced fluorescence (SIF) using two open-source R packages [2,3] (i.e., FieldSpectroscopyDP and FieldSpectroscopyCC), available at: https://github.com/tommasojulitta/FieldSpectroscopyDP and https://github.com/tommasojulitta/FieldSpectroscopyCC. Daytime periods*:* Data were collected continuously the whole day from 1 AM and 9 PM (GMT/UTC) resulting in approximately 1000 measurements per day. For your information: The midnight sun period lasts from April 20th to August 23rd in Longyeabyen/Adventdalen.
Data source location	The data were collected at a high-Arctic site in Adventdalen, a large periglacial valley on Svalbard; Norway (78.1853 N, 15.92518 E).
Data accessibility	Repository name: The Arctic Data Centre of the Norwegian Meteorological Institute, Oslo; Norway (adc.met.no). CC-BY-4.0. Norwegian Meteorological Institute, Free Meteorological Data | re3data.org. re3data.org: Norwegian Meteorological Institute, Free Meteorological Data; editing status 2021-07-12; re3data.org – Registry of Research Data Repositories. https://doi.org/10.17616/R3GS5Z last accessed: 2023-07-19. Data identification number: https://doi.org/10.21343/ZDM7-JD72 Direct URL to data: https://thredds.met.no/thredds/catalog/arcticdata/infranor/NINA-FLOX/raw/catalog.html; https://doi.org/10.21343/ZDM7-JD72 under a CC-BY-4.0 license. [4] Tømmervik, H., & Nilsen, L. (2023). SIOS instrument #49 - Hyperspectral measurements including sun-induced fluorescence (SIF) in Adventdalen. Norwegian Meteorological Institute. https://doi.org/10.21343/ZDM7-JD72

## 1 Value of the Data

• The data are useful and important for the calibration of existing data on phenology on Svalbard (Svalbard Integrated Observation System - SIOS), existing networks of Eddy-Covariance sites (FLUXNET) and Arctic Report Card (ARC). The data may advance the knowledge between the diurnal and seasonal dynamics of canopy chlorophyll fluorescence and reflectance, vegetation indices (VIs), Solar-Induced Fluorescence (SIF), and gross ecosystem productivity (GPP).
• The data may benefit ecologists, zoologists, botanists, and plant physiologists, by providing inputs for models and processes to demonstrate the connections between reflectance and fluorescence properties for monitoring vegetation photosynthetic function and detecting climatic stress.*)*.
• The data can be used by remote sensing researchers for calibration and validation of canopy VIs, reflectance, and SIF signals of High Arctic tundra vegetation currently measured by satellite instruments (i.e. ENMAP, GOME-2, GOSAT, OCO-2, Sentinel-2/3, Landsat, MODIS/VIIRS, etc.).
• Such data may provide important information of habitat fertility for the Svalbard reindeer *(Rangifer tarandus* subsp*. platyrhynchus),* which is a climatic sensitive sub-species of the reindeer/Caribou *(Rangifer tarandus).*

## 2 Data Description

Hyperspectral sensors are widely used in monitoring of phenology and spectral properties of vegetation [5]. Hyperspectral sensors normally measure hundreds of spectral bands, 600 or more, with bandwidths usually less than 10 nm down to sub-nanometer level. When installed in the field, these sensors typically measure reflected light from a target. If the reflected radiation is, then normalized by the downwelling light flux it provides the reflected spectral signatures of vegetation [6]. We have in this study used a hyperspectral instrument, FloX (JB Hyperspectral Devices GmbH, Düsseldorf, Germany) which is a field spectrometer designed for continuous and long-lasting high-resolution spectral measurements of radiances, particularly for the Sun-Induced Fluorescence (SIF) at the top-of-canopy [1]. SIF is an optical signal emitted in the spectral range 650–850 nm from chlorophyll-a molecules in vegetation [7] and it is closely related to photosynthetic activity as light absorbed by chlorophyll in leaves is used for photochemistry, non-photochemical quenching (NPQ) and fluorescence [8]. Thus, it has been increasingly used as a proxy for gross primary productivity (GPP) as well as carbon flux estimation. The continuous SIF data acquired by the FloX-system can be good proxies for the measured carbon fluxes by EddyCovariance towers and are important ground validation measurements sites for satellite data [6,9]. Land surface observations from near-ground instruments including (PhenoCam, NDVI sensors, etc.) and hyperspectral sensor systems like FLoX remain essential to fill spatial and temporal gaps in satellite acquisition of cloud-free data [9] and to correctly interpret remotely sensed parameters (indices) to actual changes in ecosystem functioning, composition, and phenological stages [9,10]. The processed data (all_index_2019_2020_2021_2022/) are accessible at: https://thredds.met.no/thredds/catalog/arcticdata/infranor/NINA-FLOX/raw/catalog.html.

The processed files and contain data from the starting year of measurement until year 2021 (https://thredds.met.no/thredds/catalog.html). The following parameters are available in the dataset : the number day of the year fraction (doy.dayfract), datetime [UTC], incoming radiation at 750 nm [W m-2 nm-1sr-1], reflected radiation at 750 nm [W m-2 nm-1sr-1], dynamic range covered of the upward channel in percent (dynamic range E [%]), Sun Induced Fluorescence - SIF_A_ifld [mW m-2 nm-1sr-1], dynamic range E full [%], and common indices like Normalized Difference Vegetation Index (NDVI), photochemical reflectance index (PRI), enhanced vegetation index (EVI) (see Table 1 for full description of the dataset).

**Table 1:** Variable/acronym name, description and unit for the derived variables available in the catalog: https://thredds.met.no/thredds/catalog/arcticdata/infranor/NINA-FLOX/raw/all_index_2019_2020_2021_2022/catalog.html.

Variable name	Description	Units
doy.dayfract datetime SZA Lat Long temp1 temp2 temp3 temp4 Incoming at 750nm Reflected 750 Reflected 760 Reflected 687 Reflectance 750 Reflectance 760 E_stability sat value L [boolean] sat value E [boolean] sat value E2 [boolean] Dynamic range E Dynamic range L SIF_A_ifld SIF_B_ifld Incoming at 750 nm Full Reflected 750 nm Full PAR ref PAR APAR E_stability full sat value L full [boolean] sat value E full [boolean] sat value E2 full [Boolean] Dynamic range E full Dynamic range L full SSHIFT Vegetation indices	Day of the year and day fraction Date time, format UTC Solar Zenith Angle Latitude of observation Longitured of observation Temperature of QEPro CCD Temperature of QEPro housing Temperature of FloX mainboard Temperature of FloX spectrometer compartment Incoming radiance at wavelength 750 nm Reflected radiance at wavelength 750 nm Reflected radiance at wavelength 760 nm Reflected radiance at wavelength 687 nm Reflectance at wavelength 750 nm Reflectance at wavelength 760 nm Percentage difference between WR1 and WR2. Fluo range Saturation value of downward channel. Fluo range Saturation value of upward channel 1. Fluo range Saturation value of upward channel 2. Fluo range Dynamic range covered of upward channel. Fluo range Dynamic range covered of downward channel. Fluo range SIF at O2A band (760 nm). Ifld method SIF at O2B band (687 nm). iFLD method Incoming radiance at wavelength 750 nm. Full range Reflected radiance at wavelength 750 nm. Full range Photosynthetically active radiation Reflected photosynthetically active radiation Absorbed photosynthetically active radiation Percentage difference between WR1 and WR2. Full range Saturation value of downward channel. Full range Saturation value of upward channel 1. Full range Saturation value of upward channel 2. Full range Dynamic range covered of upward channel. Full range Dynamic range covered of downward channel. Full range Spectral shift of Full range spectrometer Various VIs defined in indices (Table 2)	Daynumber Format UTC Degrees Degrees Degrees Degrees Degrees Degrees Degrees W m-2 nm-1sr-1] W m-2 nm-1sr-1] W m-2 nm-1sr-1] W m-2 nm-1sr-1] Ratio [0–1] Ratio [0–1] % % % mW m-2 nm-1sr-1 mW m-2 nm-1sr-1 W m-2 nm-1sr-1 W m-2 nm-1sr-1 W m-2 μmol m-2 s-1 μmol m-2 s-1 % % % nm Ratio

The explanation of the different vegetation indices presented in this paper is calculated and reported according to names/types, formulas, and references (Table 2). To calculate fluorescence, radiance, reflectance, and a variety of vegetation indices, an open-source R package is available on GitHub platform at https://github.com/tommasojulitta, and released under the license GNU v3.0. The package is wrapped with a graphical user interface to allow easy processing of data.

**Table 2:** Vegetation indices - formula and reference. R = Red band; G = Green band, B = Blue band; NIR = Near Infrared band (NIR); RE1 = Red edge band #1; RE2 = Red edge band #2; *ρ =* specific band.

Index	Name	Formula	Reference and site
NDVI	Normalized Difference Vegetation Index	NDVI = (NIR − R)∕(NIR + R)	Rouse et al. (1974) https://ntrs.nasa.gov/citations/19740022614
PRI	Photochemical Reflectance Index	PRI = (*ρ*570 − *ρ*531)/*ρ*570 + *ρ*531)	Gamon et al. (1992) https://www.sciencedirect.com/science/article/abs/pii/003442579290059S
MTCI	MERIS Terrestrial Chlorophyll Index	MTCI = (NIR − RE1)∕(RE1 − R)	Dash and Curran (2004) https://www.tandfonline.com/doi/abs/10.1080/0143116042000274015
SR	Simple Ratio	SR = NIR/Red	Jordan (1969); https://esajournals.onlinelibrary.wiley.com/doi/abs/10.2307/1936256
rep	Red edge position	REP = *ρ*700 + 40[(*ρ*670 + *ρ*700/*ρ*740 − *p*700)]	Clevers et al. (2002). https://www.tandfonline.com/doi/abs/10.1080/01431160110104647
NIR_V_	Near-Infrared Reflectance of vegetation index	NIRv = *ρ*_NiR_	Turner et al. (2020). https://bg.copernicus.org/articles/17/405/2020/
EVI	Enhanced Vegetation Index	EVI = 2.5[(NIR − R)/(NIR + 6 × R − 7.5 × B + 1)]	Huete et al. (2002) https://www.sciencedirect.com/science/article/pii/S0034425702000962
TCARI	Transformed Chlorophyll Absorption Reflectance Index	TCARI = 3[(RE1 − R) − 0.2(RE1 − G)(RE1∕R)]	Haboudane et al. (2002) PII: S0034-4257(02)00018-4 (sciencedirectassets.com)
REDCL	Red edge chlorophyll index	REDCL = NIR∕RE1 − 1	Gitelson et al. (2005) https://agupubs.onlinelibrary.wiley.com/doi/full/10.1029/2005GL022688
MCRI	Modified Chlorophyll Absorption Ratio Index (MCARI)	MCRI = [(RE2 − RE1) −0.2(RE2 − R) × RE2∕RE1]	Daughtry et al. (2000) https://www.sciencedirect.com/science/article/pii/S0034425700001139

The objective of this paper is to describe a three-year hyperspectral dataset (2019–2021) from a high-Arctic site in Adventdalen, a large periglacial valley on Svalbard (78.2 N, 15.9 E) experiencing large climatic change [11]. This paper specifies how the data was collected by the sensor and processed, and briefly discusses how this sensor can be used as a calibration point for analysis of satellite data and replacement for satellites under cloudy conditions. This dataset will be continuously updated over the next 6 years (every year) following the SIOS work plan [12].

## 3 Experimental Design, Materials and Methods

### 3.1 Site description

**Fig. 1:**
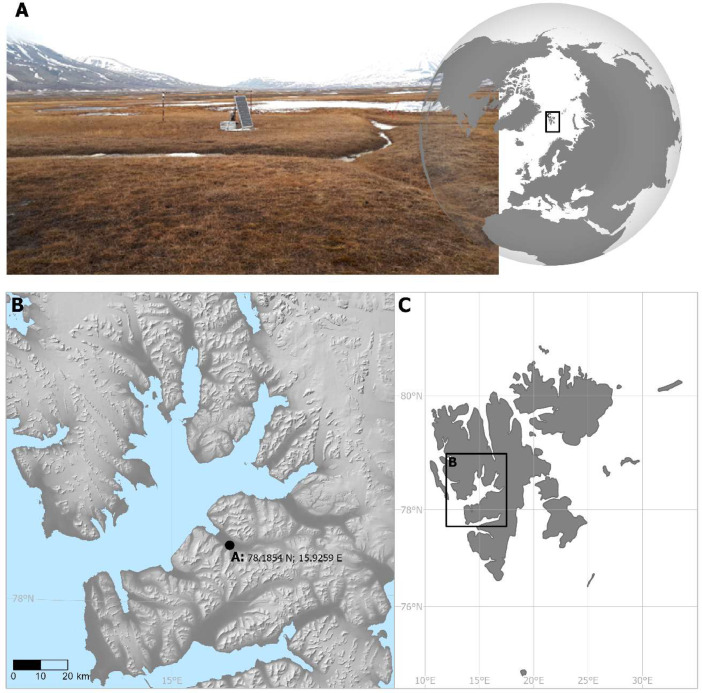
The FloX-spectrometer is station established in Adventdalen, Spitsbergen, Svalbard, 78.1854 N; 15.9259 E. The vegetation is dominated by the grass *Dupontia fisheri*, while the graminoid *Eriophorum scheuchzeri* is subdominant (A). The location is indicated in the insets (B) and (C).

The site is located close to and within the footprint of the EC tower [13] in Adventdalen (Fig. 1). The vegetation composition in the valley is dominated by the dwarf shrub species *(Salix polaris, Cassiope tetragona* and *Dryas octopetala*), herbs and grasses like *Eriophorum scheuchzeri, Luzula confusa, Alopecurus ovatus, Dupontia fisheri* and *Poa* spp. [10]. The species distribution differs with surface wetness, which is mostly governed by the microtopography. At the site, the vegetation is dominated by graminoids on a silty-sandy plain characterized by large scale polygon cryoturbation [13]. Vegetation cover in the footprint of the spectrometer is 100 % and dominant vascular species are *Dupontia fisheri* and *Eriophorum scheuchzeri* [10]. The terrain is gently sloping towards the Adventdalen river.

### 3.2 Instrumentation

FloX is a field spectrometer designed for continuous and long-lasting high-resolution spectral measurements of upwelling and downwelling radiances according to the manufacturer's user manual. It measures spectral data of extremely high spectral resolution (less than a nanometer) and is designed to work continuously under variable outdoor environments. SIF is a radiation flux emitted by chlorophyll molecules in the red (RSIF) and far-red regions (FRSIF) and provides a better proxy for photosynthesis independent of ancillary information or modeling steps which is needed by other methods [1,14]. The FloX is equipped with two grating spectrometers: (i) QEPro (Ocean Optics, Largo FL, USA) with high spectral resolution (FWHM∼0.3 nm: SSI∼0.15 nm) in the fluorescence emission range 650–800 nm: (ii) FLAME S (Ocean Optics, Largo FL, USA) covering the full range of VIS-NIR (FWHM∼1.7 nm; SSI∼0.6 nm).

**Fig. 2:**
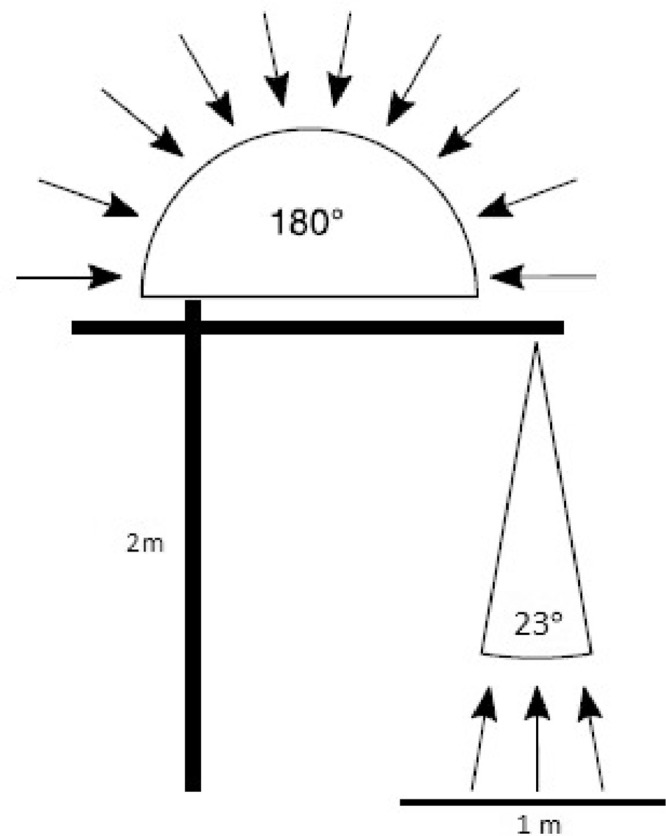
Light reception (incoming radiation) of the cosine receptor of the upward looking fiber with a hemispherical field of view (180°). The downward looking bare fibers mounted 2 m above the vegetation have a field of view of 23° (Figure based on a drawing by JB-Hyperspectral Devices).

The spectometer's entrance is split towards two optical fibers (Fig. 2) that lead to a cosine receptor measuring the downwelling (incoming) radiance with a hemispherical field of view (180°), and a bare fiber with an opening angle of 23° measuring the upwelling radiance from the canopy/vegetation with a footprint of 1 m [6].

When FloX instruments are set to automatic mode, measurements are collected continuously (or at a user predefined time interval). The integration time is automatically optimized according to the light illumination condition thus the measurements speeds is not constant, ranging in between 20 s and 60 s for each measurement cycle (upwelling, downwelling radiance and spectrometer dark current). The FloX-instrument was set to operate between 1 AM and 9 PM resulting in approximately 1000 measurements per day.

### 3.3 Data processing

**Fig. 3:**
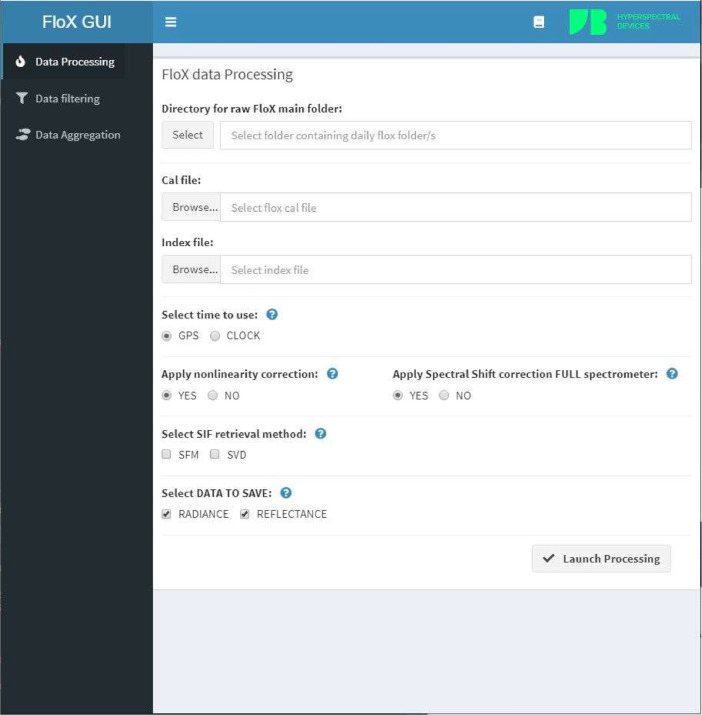
The FloX data processing GUI which is used for processing, extraction of different parameters and indices, data filtering and temporal aggregation.

The FloX system collects upwelling (reflected light from the target) and downwelling (solar light) radiation fluxes, which are processed to radiance, reflectance, and SIF using a graphical user interface (GUI, example shown in Fig. 3) made available for users of devices from JB Hyperspectral Devices [1]. The GUI is entirely based on open-source R software. The main functionalities of the GUI rely on two R packages (FieldSpectrocospyCC and FieldSpectrocospyDP [2,3]. Data filtering of the measurements is facilitated by using the related functionality of the GUI and based on quality flags, reporting information related to the illumination stability during the measurement cycle, spectra saturation, and measurements collected at solar zenith angle higher than 85°, where the cosine response of the cosine diffuser could lead to significant error in downwelling radiance. Data processing outputs are upwelling and downwelling radiance, target reflectance, apparent reflectance, red and far-red fluorescence (iFLD and SFM retrieval applied), and desired spectral indices (vegetation indices) are calculated in the FloX spectral range (400–950 nm). The SIF estimates are spectral wavelengths associated with 1) atmospheric oxygen absorption features centered at 683 (SIF B) and 2) 760 nm (SIF A). SIF A and SIF B were retrieved by applying the Fraunhofer Line Discriminator method (version 3, iFLD) and the Spectral Fitting Method (SFM) [15]. Total SIF A + B was calculated as the sum of SIF A and SIF B [1,15].

In Table 2, the definitions of the different vegetation indices presented in this paper is and reported according to formulas. To calculate fluorescence, radiance, reflectance and a variety of vegetation indices, an open-source R package is available on GitHub platform at https://github.com/tommasojulitta, and released under the license GNU v3.0. The package is wrapped with a graphical user interface to allow easy processing of data.

### 3.4 Dataset overview – examples of measurements and results from processing

**Fig. 4:**
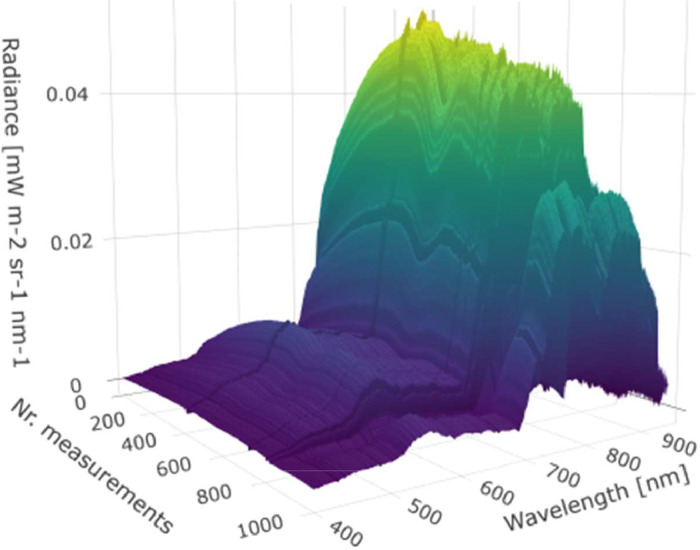
Example of daily measurements of radiances from the FLoX system (data from July 27th 2020). Number of measurements represents the number of spectral data collected during the diurnal course from 1 am to 9 pm.

**Fig. 5:**
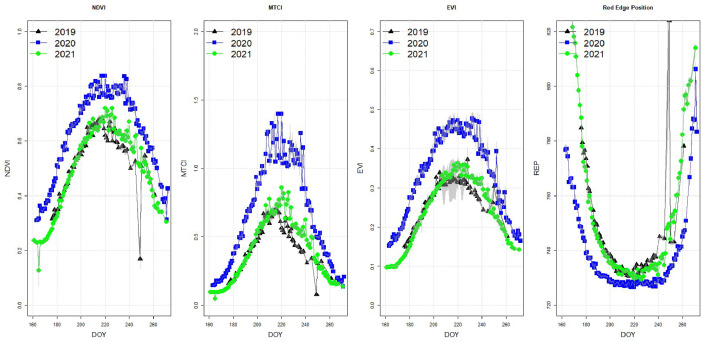
Annual Measurements of different parameters (NDVI, MTCI, EVI, Red edge position) extracted from the FLoX system for the growing seasons of 2019, 2020 and 2021.

**Fig. 6:**
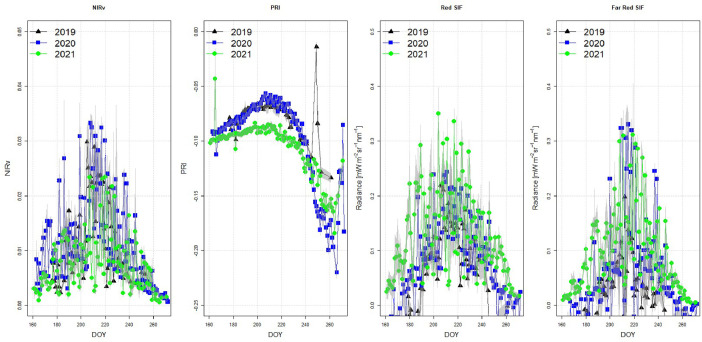
Annual measurements of NIRv, PRI, Red SIF and Far-Red SIF extracted from the FLoX-system for 2019, 2020 and 2021.

The database currently consists of data from 4 continuous growing season in the period 2019–2022, and here we present results from the first three years (2019, 2020, 2021). Fig. 4 shows an example of diurnal harvest of radiances during a single day of measurements (July 27th 2020). In Fig. 5, we present annual measurements of NDVI, MERIS Terrestrial Chlorophyll Index (MTCI), EVI, and Red Edge position (REP). In Fig. 6, we present annual measurements of sun induced fluorescence (Red SIF and Far-Red SIF), and the vegetation indices NIRv and PRI extracted from the FLoX-system for 2019, 2020, and 2021.

Collecting time series of hyperspectral data from FLoX system at high latitude environments is extremely challenging caused of climatic constraints especially at the end of the autumn season (September–October) due to reduced sunlight combined with decreasing air temperatures. This is crucial for sun power panels giving electricity to the FLoX-system. However, data collected the first years are invaluable to understand how tundra vegetation responds to changing climate and environment. Potential applications which can be explored using this dataset are (a) developing and upscaling FLoX-based SIF-GPP relations for estimating regional tundra vegetation productivity and carbon flux, (b) modeling fluorescence radiative transfer process in tundra vegetation, (c) detecting tundra vegetation phenology and identifying underlying controlling factors. The listed potential activities will naturally incorporate available data from Sentinel 2/3, MODIS, ENMAP, and OCO-2, and the nearby Eddy Covariance tower [13] and comparing them with these FLoX-data.

## 4 Limitations

The spectrometer broke down during the first two weeks of season #1 (2019) due to a short circuit, hence data was not collected during this period. It should be noted that analysis of the data at the start of the 2019 season should be considered with care.

## 5 Ethics Statement

The authors declare that the work did not involve the use of human subjects, animal experiments and information collected from social media platforms.

## CRediT Author Statement

**Hans Tømmervik:** Conceptualization, Fieldwork, Methodology, Formal analysis, Writing, review & editing; **Tommaso Julitta:** Software, Methodology, Formal analysis, Writing, review & editing*;* **Lennart Nilsen**: Conceptualization, Fieldwork, Writing, review & editing; **Taejin Park:** Formal analysis, Writing, review & editing***;* Andreas Burkart:** Data curation, Writing, review & editing; **Katarzyna Ostapowicz:** Fieldwork, Writing, review & editing**; Stein Rune Karlsen:** Writing, review & editing**; Frans-Jan Parmentier:** Writing, review & editing; **Norbert Pirk**: Fieldwork, Writing, review & editing; **Jarle W. Bjerke:** Writing, review & editing.

## Data Availability

https://thredds.met.no/thredds/catalog/arcticdata/infranor/NINA-FLOX/catalog.html (Original data) (Norwegian Meteorological Institute).
